# Wildlife-derived *Lactobacillus agilis* LA-V4 alleviates *Salmonella* Typhimurium infection in broilers through gut microbiota remodeling and hepatic autophagy restoration

**DOI:** 10.1016/j.psj.2026.107661

**Published:** 2026-08-19

**Authors:** Chuxian Quan, Siyuan Li, Ziyang Wang, Rui Zhao, Muhammad Farhan Rahim, Mengdi Zhang, Chao Jin, Haofang Yuan, Yan Li, Shan Wu, Saisai Gong, Quan Mo, Jiakui Li

**Affiliations:** a College of Veterinary Medicine, Huazhong Agricultural University, Wuhan, 430070, China; b College of Animal Science and Technology, Tarim University, Alar, 843300, China; c College of Veterinary Medicine, Sichuan Agricultural University, Chengdu, 611130, China

**Keywords:** *Lactobacillus agilis*, *Salmonella* Typhimurium, Gut microbiota, Hepatic autophagy

## Abstract

*Salmonella* Typhimurium (*S.* Typhimurium) is a major enteric pathogen in poultry production, causing substantial economic losses and posing a serious public health threat through the contamination of animal-derived food products. Although probiotics are increasingly recognized as promising alternatives to antibiotics for controlling *Salmonella* infection, their mechanistic basis of protection remains insufficiently defined. Here, we evaluated the probiotic efficacy of *Lactobacillus agilis* LA-V4, a stress-tolerant strain isolated from vulture feces, in healthy Arbor Acres broilers and in a prophylactic model of *S.* Typhimurium infection. In healthy birds, LA-V4 supplementation enhanced mucosal immune responses and appeared to alleviate hepatic metabolic stress associated with rapid early growth. In *S. Typhimurium*-challenged broilers, LA-V4 markedly improved intestinal barrier integrity by upregulating the tight junction proteins ZO-1 and Occludin. Moreover, LA-V4 pretreatment was associated with a significantly lower relative abundance of *Salmonella*-assigned sequences in the cecal microbiota and partially ameliorated infection-associated microbial dysbiosis, as evidenced by decreased relative abundances of Firmicutes-related taxa, such as Lachnospiraceae and Erysipelotrichales, together with increased relative abundances of *Lactobacillus* and several putative short-chain fatty acid-producing taxa, including v9d2013_group, *Phascolarctobacterium*, and *Fournierella*. In hepatic tissue, LA-V4 restored autophagic activity, as reflected by increased LC3-II and Beclin1 expression and reduced p62 accumulation. Taken together, our findings identify *L. agilis* LA-V4 as a novel probiotic candidate that protects broilers against *S.* Typhimurium infection through coordinated regulation of the gut microbiota, intestinal barrier function, and hepatic autophagic responses.

## Graphical abstract

**Figure:**
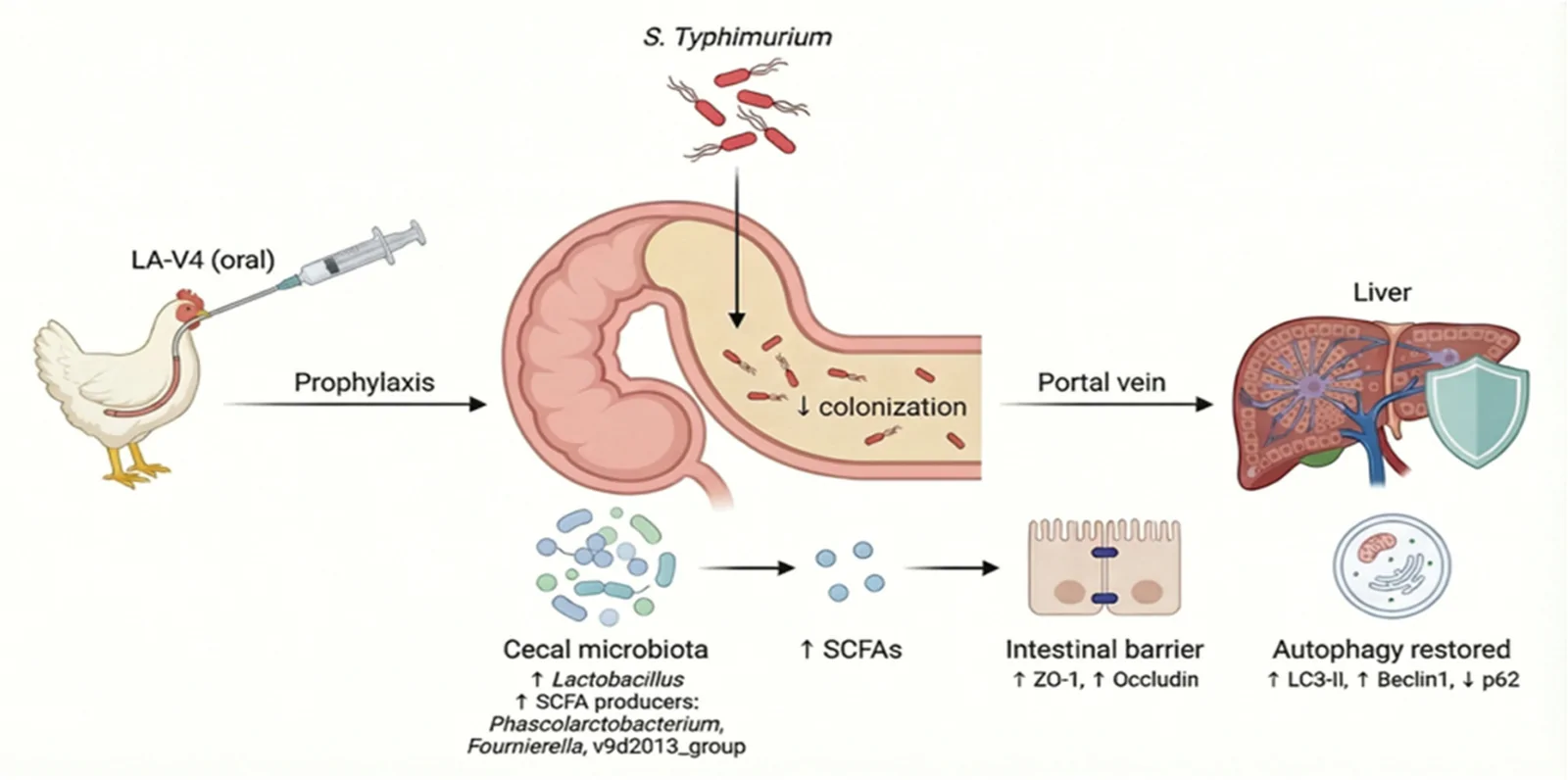

## Introduction

*Salmonella* is a facultative intracellular pathogen capable of infecting humans and a wide range of animal species (Zhang et al., 2008). Among the numerous serovars, *Salmonella enterica* serovar Typhimurium (*S*. Typhimurium) is one of the major pathogens associated with salmonellosis in poultry and humans. In broilers, *S*. Typhimurium can colonize the intestinal mucosa, disrupt epithelial barrier integrity and gut microbial homeostasis, and subsequently disseminate to systemic organs, thereby impairing growth performance and increasing mortality, particularly in young birds (Fasina, et al., 2008; Oastler, et al., 2022). Moreover, *S*. Typhimurium can manipulate host autophagy to promote intracellular survival and systemic dissemination, representing an important immune-evasion strategy (Ganesan, et al., 2017). Although vaccination and antibiotics are widely used for prevention and control, incomplete vaccine protection, antimicrobial resistance, and concerns regarding drug residues limit their effectiveness (Qiao, et al., 2018; Vandeplas, et al., 2010). Therefore, safer and more effective non-antibiotic strategies are urgently needed to control S. Typhimurium infection in poultry.

Probiotics are beneficial microorganisms that can improve growth performance, modulate immune responses, and maintain gut microbial homeostasis in poultry (Abd El-Hack, et al., 2022). Supplementation during the early growth stage has been shown to enhance growth and optimize the gut microbiota of broilers, while also improving resistance to bacterial pathogens such as Escherichia coli (Abdel-Shafi, et al., 2023; Agustono, et al., 2022; El-Sharkawy, et al., 2020; Vineetha, et al., 2017; Yin, et al., 2024). Moreover, probiotic supplementation has been reported to alleviate intestinal injury and inflammatory responses in broilers challenged with *S*. Typhimurium (Lou, et al., 2024). These findings support the potential application of probiotics as non-antibiotic strategies for controlling enteric bacterial infections in poultry.

Lactic acid bacteria (LAB) are common and abundant members of the human and animal gut microbiota. Growing evidence suggests that lactic acid bacteria (LAB) can establish themselves in the immature gut of young animals, thereby contributing to protection against early-life infections (Li, et al., 2024; Maldonado, et al., 2018). Among LAB, lactobacilli have received extensive attention because of their probiotic potential. For instance, *Ligilactobacillus salivarius* has been shown to alleviate inflammatory responses induced by enterotoxigenic *Escherichia coli* (ETEC) in *vitro* (Qiao, et al., 2020). Similarly, *Lactobacillus johnsonii* L531 demonstrated the ability to attenuate the oxidative damage associated with *S.* Typhimurium-induced diarrhea in mice (Chen, et al., 2023). More importantly, previous studies tracking the establishment of lactic acid bacteria in neonatal feces during the first week of life have shown that *Ligilactobacillus agilis* (*L. agilis*) can become established in the neonatal gut at an early stage (Al-Balawi and Morsy, 2020). Accumulating evidence indicates that *L. agilis* exhibits promising probiotic potential, including antibacterial activity and intestinal anti-inflammatory effects (Khan, et al., 2021; Shi, et al., 2021). For example, L. agilis SNF7 was reported to alleviate E. coli-induced diarrhea in mice, accompanied by marked antibacterial and anti-inflammatory effects(Feng, et al., 2024). Although these studies suggest that *L. agilis* may inhibit intestinal pathogens and support gut health, probiotic efficacy is highly strain specific. In our preliminary work, we isolated a probiotic strain from vultures, designated as *Lactobacillus agilis* LA-V4 (*L. agilis* LA-V4). In *vitro* assays demonstrated that LA-V4 exhibited strong antagonistic activity against multiple bacterial pathogens and maintained high viability under simulated gastrointestinal conditions (Li, et al., 2026). However, whether it can translate these in vitro characteristics into comparable protective effects in vivo remains largely unknown. Therefore, in the current study, we initially evaluated the effects of dietary LA-V4 supplementation on the growth performance and intestinal barrier function of healthy broilers. Subsequently, we established an *S.* Typhimurium challenge model to systematically investigate the modulatory effects of prophylactic LA-V4 treatments of growth measures, intestinal morphology, mucosal integrity of barrier, and cecal microbiota in infected chicks. Ultimately, this study aims to provide a novel and efficacious microecological intervention strategy for mitigating *S*. Typhimurium infections in the poultry industry.

## Materials and methods

### Chemicals and reagents

LB and MRS broths were purchased from Qingdao Hope Bio-Technology Co., Ltd. Chicken-specific ELISA kits for sIgA, IgM, and IgG were obtained from Baolai Biotechnology, while assay kits for chicken IL-1β, IL-6, and TNF-α were provided by Junxing Biological Technology. Antibodies against LC3, p62, Beclin1, Occludin, and β-actin, as well as the HRP goat anti-rabbit IgG, were all purchased from Wanleibio Co., Ltd. (Shenyang, China), while the ZO-1 primary antibody was purchased from Proteintech Group, Inc. (Wuhan, China). All serum biochemical assay kits were supplied by Shenzhen Mindray Animal Medical Technology Co., Ltd.

### Bacterial strains and culture conditions

*Ligilactobacillus agilis* LA-V4 used in this study was previously isolated from freshly voided fecal samples collected from apparently healthy vultures and characterized by our laboratory (Li, et al., 2026). The vultures showed no obvious clinical signs of disease at the time of sampling, and detailed information regarding the sampling conditions and strain isolation procedures has been reported in our previous study. Whole-genome sequencing and genome-informed safety assessment of LA-V4, including screening for potential virulence factors and antimicrobial resistance-associated genes, were also performed in our previous study. The *Salmonella* Typhimurium strain used in this study was originally isolated from clinical samples and has been previously applied in an avian infection model in our laboratory (Wang, et al., 2026). For preparation of the bacterial inocula, the *L. agilis* LA-V4 and Salmonella strains were cultured in de Man, Rogosa, and Sharpe (MRS) broth and Luria-Bertani (LB) broth, respectively. The *L. agilis* LA-V4 and *Salmonella* strain was incubated at 37°C in a shaking incubator. After approximately 12 hours of incubation, the bacterial cultures were harvested by centrifugation (5000 rpm for 5 min). The supernatants were discarded, and the resulting bacterial pellets were washed with sterile physiological saline (0.9% NaCl). Finally, the bacterial suspensions were resuspended in sterile saline and adjusted to a final concentration of approximately 1.0 × 10⁸ CFU/mL for subsequent experimental use.

### Broiler’s management and experimental design

Day-1-old Arbor Acres (AA) broilers were obtained from Chia Tai Poultry Co., Ltd. (Xiangyang, China) and housed in the standardized animal facility at the Animal Experiment Center of Huazhong Agricultural University. The light/dark cycle was kept at 12-h light/12-h dark with a relative humidity of 50%55 in the housing environment. The ambient temperature was kept at 36±2°C and was gradually changed depending on the normal growth cycle of a broiler and it was relatively steady in each stage of development. A basic commercial starter diet was given to all experimental birds and ad libitum access to feed and water was offered. Before the experimental challenge, the birds were not specifically screened for Salmonella by bacteriological culture or PCR. However, no clinical signs of enteric disease, diarrhea, or abnormal mortality were observed during the acclimatization and pre-challenge periods. A randomized controlled trial was used to examine the prophylactic effect of early *Lactobacillus agilis* LA-V4 supplementation on intestinal health and the Salmonella-induced diarrhea. The chicks were randomly allocated into 5 groups (n = 15) to evaluate two experimental modules: Experiment-I consisted of a Control group (Con) and an LA-V4 supplementation group (LA). Following a 3-day acclimatization period, chicks in the Control group were administered 1 mL of 0.9% sterile saline daily via oral gavage, while chicks in the LA group were orally gavaged with an equal volume of the LA-V4 suspension (1 × 10⁸ CFU/mL). Experiment-II focused on the *Salmonella* challenge and comprised a Control group (Con), a *Salmonella*-infected group (SA), and an LA-V4 pre-treated + *Salmonella*-infected group (LASA). After the 3-day acclimatization, the SA and Con groups were orally gavaged with 1 mL of 0.9% saline daily, whereas the LASA group received an equal volume of the LA-V4 suspension (1 times 10^8^ CFU/mL) daily. LA-V4 was administered only during the prophylactic supplementation period and was discontinued at the time of the *S*. Typhimurium challenge; no further LA-V4 was administered after challenge. On day 14, birds in the SA and LASA groups were challenged via oral gavage with 1 mL of *Salmonella* Typhimurium suspension (1 times 10^8^ CFU/mL). The Con group received an equal volume of sterile saline concurrently. Birds were observed rigorously after infection up to day 21. The animal experimental protocol was reviewed and approved by the Animal Care and Welfare Committee of Huazhong Agricultural University (approval No. 202601270035), and all procedures were conducted in accordance with the institutional guidelines for the care and use of laboratory animals.

### Sample collection and storage

All the biological samples were collected in a systematic manner at the stipulated endpoint of the experiment. The blood was first collected by drawing it into the standard blood collection tubes through the jugular vein and then the broilers were euthanized through cervical dislocation. Representative sections of liver and jejunum were immediately removed on necropsy and fixed in 4% paraformaldehyde to be subjected to histopathological tests later. The rest of these tissues were snap-frozen and kept at −80°C to conduct additional molecular analyses. Also, cecal samples were collected aseptically into sterile microcentrifuge tubes and frozen at −80°C until further use.

### Histopathological analysis and morphometric evaluation

Tissue samples (jejunum and liver) were washed carefully with ice-cold phosphate-buffered saline (PBS) to clear intestinal contents and residual blood and then fixed at room temperature in 4% paraformaldehyde to fix them at least 24 h. After fixation, the specimens were dehydrated by a series of graded ethanol (70%, 80%, 90%, 95% and 100%), cleared in xylene, and embedded in paraffin. The tissue blocks embedded in paraffin were cut into 4-5 μm thick serial sections with the help of a rotary microtome (RM2016, Leica, Wetzlar, Germany). The sections that were obtained were placed on glass slides, deparaffinized with xylene, rehydrated using progressively diluted solutions of ethanol and stained with hematoxylin and eosin (H&E) routinely. Finally, the histopathological changes in the jejunum and liver were examined under a biological light microscope (BX53, Olympus, Tokyo, Japan). A digital slide scanner (Pannoramic MIDI, 3DHISTECH, Budapest, Hungary) was then used to scan the slides to obtain representative high-resolution scans. CaseViewer 2.4 software was used to measure morphometric parameters of the jejunal villus height and crypt depth.

### Analysis of serum biochemical and immunological parameters

The serum biochemical parameters such as aspartate aminotransferase (AST), alanine aminotransferase (ALT), creatine kinase (CK), creatinine (CREA), uric acid (UA), and triglycerides (TG) were measured using an automated veterinary biochemical analyzer (BS-240 VET, Mindray, Shenzhen. The serum concentrations of inflammatory cytokines (IL-1β 2 −6 and TNF -α) and immunoglobulins (IgM, sIgA, and IgG) were measured with the help of corresponding chicken-specific enzyme-linked immunosorbent assay (ELISA) kits to examine the systemic immune and inflammatory responses. Every ELISA test was carried out in strict compliance with the instructions of the manufacturers.

### Western blot analysis

Tissues frozen at −80°C were thawed and placed in tubes with RIPA lysis buffer (Beyotime, Shanghai, China) mixed with protease inhibitors (Beyotime). A cryogenic tissue grinder (Lixin, Shanghai, China) was used to completely homogenize the tissues. This was followed by centrifugation of the homogenates at 12, 000 rpm in 15 min at 4°C followed by the careful collection of the supernatants. The concentration of total proteins was measured with the help of a BCA Protein Assay Kit (Beyotime). All protein samples were mixed with loading buffer and denatured by boiling at 100°C for 5 min. Equal amounts of total protein (30 μg) per lane were separated by 8-15% sodium dodecyl sulfate-polyacrylamide gel electrophoresis (SDS-PAGE) and subsequently transferred onto polyvinylidene fluoride (PVDF) membranes. The skimmed milk (5%) in Tris-buffered saline (0.1%TBST) was used to block the membranes during 2 h at room temperature. After the blocking procedure, the membranes were left overnight at 4°C in the presence of the respective specific primary antibodies for 14-16 hours. The membranes were then carefully washed five times in TBST after which they were allowed to incubate with the right horseradish peroxidase (HRP)-conjugated secondary antibodies for 2 h at room temperature. Lastly, the bands of target proteins were observed with a detection kit of enhanced chemiluminescence (ECL) and scanned through an imaging system. The protein bands were densitometrically quantified with ImageJ software, with the normalization of the protein bands in ImageJ being that of β-actin as the internal reference control. Detailed information of the primary and secondary antibodies is given in Table 1.

**Table 1:** Antibodies used in Western blotting.

Target protein		Company Name
ZO-1	21773-1-AP	Proteintech Biotechnology Co., Ltd. Wuhan, China
Occludin	27260-1-AP	Proteintech Group, Inc. Wuhan, China
Beclin1	WL02237	Shenyang Wanlei Biotechnology Co., Ltd. Shenyang, China
LC3	WL01506	Shenyang Wanlei Biotechnology Co., Ltd. Shenyang, China
p62	WL02385	Shenyang Wanlei Biotechnology Co., Ltd. Shenyang, China
β-actin	WL01372	Shenyang Wanlei Biotechnology Co., Ltd. Shenyang, China

### Real-time PCR analysis

Sample tissues were frozen and then retrieved out of the −80°C freezer. The whole RNA of the homogenated tissues was isolated with RNAiso Plus (Takara, Dalian, China) following the guidelines of the manufacturer strictly. The purity and concentration of extracted RNA was measured in a NanoDrop 2000 spectrophotometer (Thermo Fisher Scientific, Waltham, MA, USA). This was followed by reverse-transcription of the RNA into complementary DNA (cDNA) with the help of a reverse transcription kit (Vazyme Biotech, Nanjing, China). The relative gene expression was determined in a 2-step technique by quantitative real-time PCR on a Real-Time PCR System (Bioer Technology, Hangzhou, China) using a particular fluorescent dye (SYBR qPCR Master Mix, Vazyme). Primer sequences of each target gene and the internal control (β- actin) are included Table 2. All samples were run in triplicate, and the relative mRNA expression levels were calculated using the standard 2^−△△Cq^ method.

**Table 2:** Primer sequences used for RT-qPCR analysis.

Gene name	Forward (5′−3′)	Reverse (5′−3′)
ZO-1	GACGGTTTCACACCTTTGGC	CGCTGCCTTCGTATCATCCT
Occludin	GACCCCCAGGAGGCTGT	ACGCTGGCCCCATCC
Beclin1	AACCAGATGCGTTATGCTC	ATTGTGCCAAACTGACCAC
LC3	GAATGTGGCAATCACTTTAC	CAGGAGGAATTTTGTCTTAG
p62	TGGAGCCGCAAGTGAATCTT	CCAGAGTAGTGGGCAAGCAA
β-actin	GAGAAATTGTGCGTGACATCA	CCTGAACCTCTCATTGCCA

### Genomic DNA Extraction, 16S rRNA gene amplification, and bioinformatics analysis

To characterize the composition of the intestinal microbial community, cecal content samples were collected, and total genomic DNA was extracted using the QIAamp DNA Stool Mini Kit (Qiagen, Hilden, Germany) according to the manufacturer’s instructions. After this the concentration and purity of the resulting DNA were accurately measured with a NanoDrop spectrophotometer, accompanied by 1% agarose gel electrophoresis which was done to confirm the integrity of fragments. Targeting the hypervariable V3-V4 region of the bacterial 16S rRNA gene, PCR amplification was performed using specific universal primers (338F: 5′-ACTCCTACGGGAGGCAGCAG-3′ and 806R: 5′-GGACTACHVGGGTWTCTAAT-3′). To ensure the reliability of the amplification data, three independent technical replicates were established for all samples. After recovery and purification using the AxyPrep DNA Gel Extraction Kit (Axygen Biosciences, Union City, CA, USA), the amplicons of each sample were pooled in equimolar ratios to construct the sequencing library. Finally, high-throughput paired-end sequencing was conducted relying on the Illumina MiSeq platform. The raw sequencing reads were initially subject to stringent quality control, denoising and paired -end mergers in the downstream bioinformatics data mining stage to obtain high-quality sequences. Thereafter, the optimized sequences were clustered into operational taxonomic units (OTUs) at a 97% sequence similarity threshold. Representative sequences from each OTU were taxonomically annotated against the SILVA 16S rRNA reference database. In the microecological community analysis, Alpha diversity (community richness and evenness) was comprehensively evaluated by calculating the Chao1 and Shannon indices. Regarding Beta diversity, this study constructed a Non-metric Multidimensional Scaling (NMDS) model based on the Bray-Curtis distance matrix to visually present the spatial separation trends of the macroscopic community structures under different treatments. Furthermore, the Linear discriminant analysis Effect Size (LEfSe) algorithm was introduced, with the logarithmic LDA score threshold set at 3.0, to precisely identify the core differential bacterial taxa (biomarkers) playing a critical differentiating role among the groups.

### Statistical analysis

All the quantitative data statistical calculations and graphical illustrations of the results obtained in this study were performed based on the use of the GraphPad Prism 9.0 software (GraphPad Software, San Diego, CA, USA). Continuous variables are uniformly expressed as the mean ± standard deviation (SD). For inferential statistical analyses, comparisons between two independent groups were performed using an unpaired Student’s t-test. Comparisons among three or more groups were conducted using one-way ANOVA, followed by Tukey’s HSD post hoc test for pairwise comparisons. Normality and homogeneity of variance were assessed before parametric testing. A value of p < 0.05 was considered statistically significant.

## Results

### LA-V4 supplementation improves growth performance and enhances intestinal barrier function

**Fig. 1:**
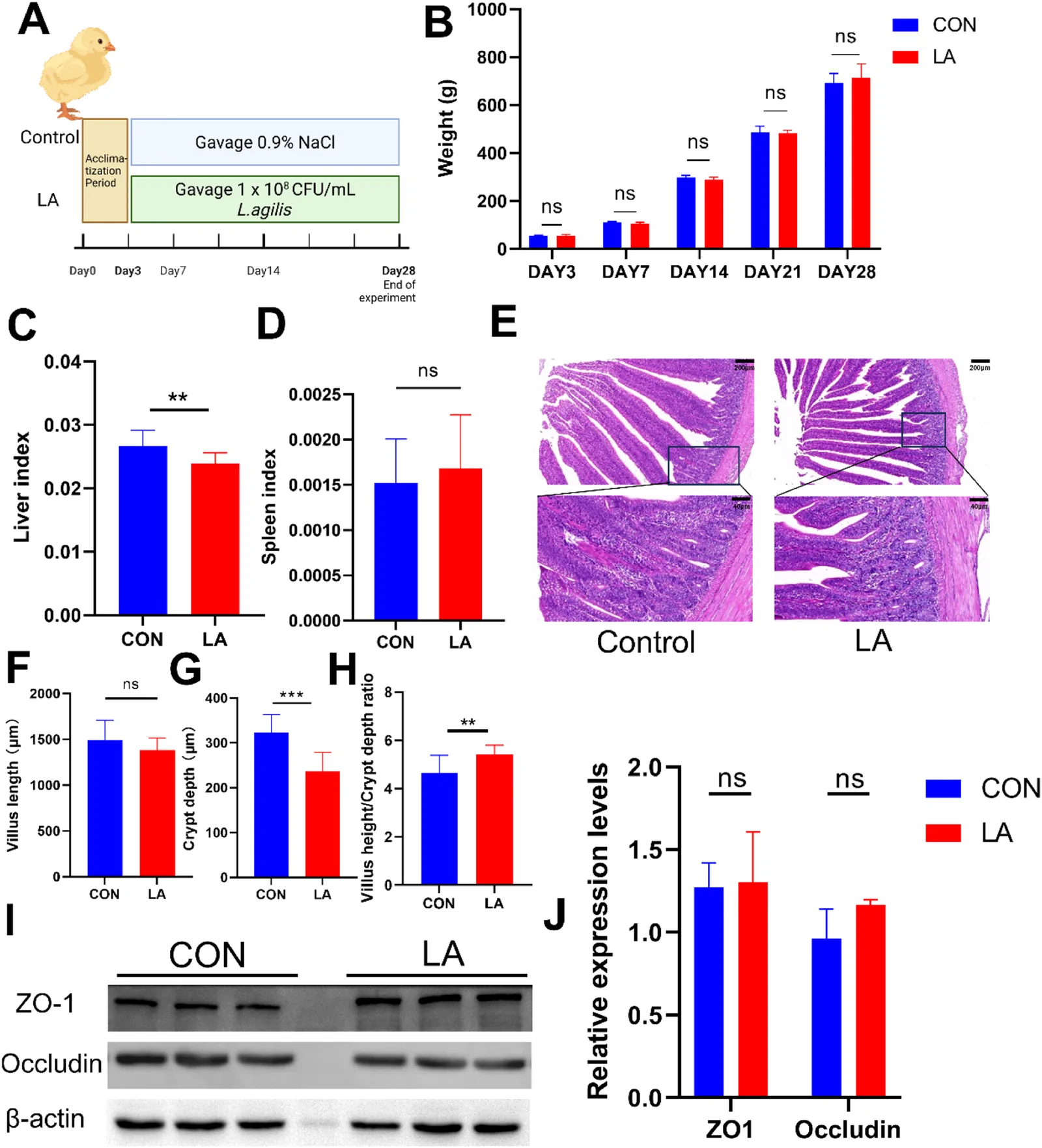
Effects of prophylactic LA-V4 supplementation on growth performance, organ indices, and intestinal barrier homeostasis in healthy broilers. (A) Schematic plan of the experimental design. (B) Body weight of broilers (C-D) Liver index and spleen index (E) Representative hematoxylin and eosin (H&E) staining images of intestinal morphology in the Control and LA groups. (F) Quantification of villus length and (G) crypt depth based on the H&E-stained intestinal sections. (H) villus height-to-crypt depth ratio (I-J) Relative protein expression levels of ZO-1 and Occludin normalized to β-actin. Data are presented as mean ± SD. **p < 0.01$, ***p < 0.001; ns, no significant difference.

To evaluate the effects of LA-V4 supplementation on intestinal health and growth performance in broilers, a 4-week in vivo trial was conducted (Fig. 1A). Continuous supplementation with LA-V4 throughout the trial produced no apparent adverse effects on broiler growth or general health. In particular, body weight did not differ significantly between the LA-V4 and control groups at any of the evaluated time points (Fig. 1B). Further evaluation of organ development revealed that the spleen index remained unaffected by LA-V4 supplementation compared with the control group (Fig. 1D, *p* > 0.05). Interestingly, dietary administration of LA-V4 led to a slight but statistically significant decrease in the liver index, implying a potential modulatory effect on hepatic metabolism (Fig. 1C, *p* < 0.01). Considering that the gastrointestinal tract serves as the principal site of action for oral probiotics, we further assessed the changes in jejunal structural integrity. Histopathological examination via H&E staining revealed that the jejunal architecture in both the Control and LA groups remained completely intact, characterized by well-arranged villi and the absence of distinct pathological lesions (Fig. 1E). Subsequent quantitative morphometric analysis indicated that, although dietary supplementation with LA-V4 had no significant effect on jejunal villus length (Fig. 1F, *p* > 0.05), it highly significantly decreased the crypt depth compared to the Control group (Fig. 1G, *p* < 0.001). Furthermore, LA-V4 supplementation significantly increased the villus height-to-crypt depth ratio (Fig. 1H, *p* < 0.05). These findings indicate that LA-V4 supplementation altered jejunal villus–crypt morphology without causing apparent disruption of intestinal architecture. Further analysis of the expression of tight junction proteins showed that LA-V4 supplementation did not significantly affect the basal expression of ZO-1 and Occludin in the jejunum (Fig. 1I-J, *p* > 0.05). Together with the preceding histopathological findings, this implies that LA-V4 can preserve the inherent homeostasis of the jejunal mucosal barrier.

### Effects of LA-V4 supplementation on systemic physiological safety, hepatorenal metabolism, and immune functions

**Fig. 2:**
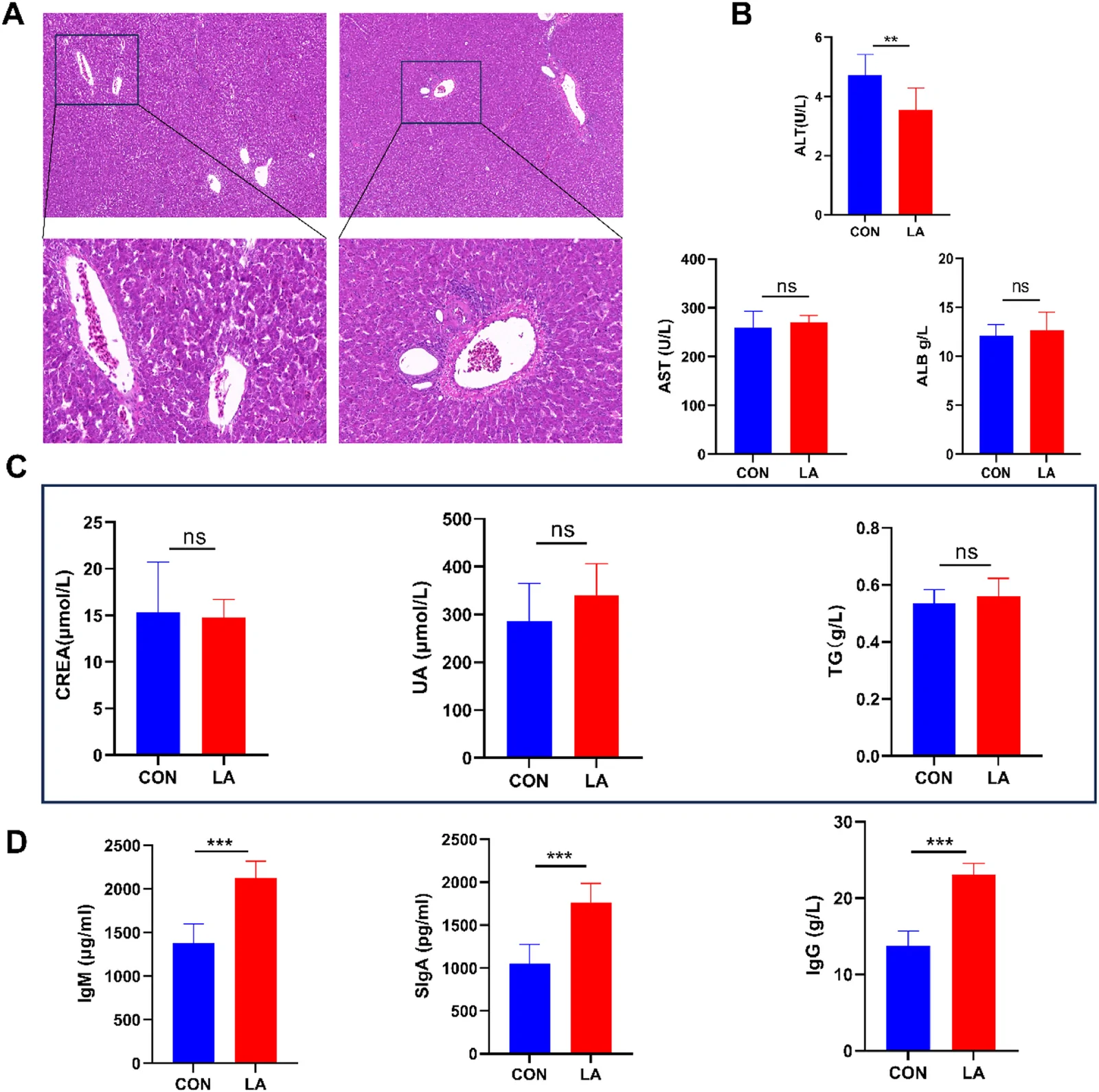
Effects of prophylactic L.A-V4 on hepatic morphology, serum biochemical indices, and systemic immunity in healthy broilers. **(A)** Representative H&E staining images of liver tissues. **(B)** Serum levels of liver function markers, including alanine aminotransferase (ALT), aspartate aminotransferase (AST), and albumin (ALB). **(C)** Serum levels of renal function and lipid metabolism markers, including creatinine (CREA), uric acid (UA), and triglycerides (TG). **(D)** Serum concentrations of immunoglobulins, including IgM, sIgA, and IgG. Data are presented as mean ± SD. **p < 0.01, ***p < 0.001; ns, no significant difference.

Based on the assessment of local intestinal homeostasis, we further assessed the impact of LA-V4 supplementation on systemic physiological functions and immune homeostasis at the histological and serological levels. As illustrated in Fig. 2A, the livers of broilers in both groups exhibited typical and healthy histological architectures, characterized by well-defined hepatic lobules, neatly arranged hepatic cords, and intact nuclear structures, without any observable pathological lesions. Serum biochemical analyses revealed that LA-V4 supplementation did not significantly alter the levels of aspartate aminotransferase (AST) and albumin (ALB) (*p* > 0.05), thereby ruling out potential hepatocellular damage and confirming the homeostasis of hepatic synthetic function. Notably, the serum alanine aminotransferase (ALT) level in the LA group was highly significantly decreased compared to the Control group (Fig. 2B, *p* < 0.01). To comprehensively evaluate the metabolic impact of LA-V4, the serum concentrations of creatinine (CREA), uric acid (UA), and triglycerides (TG) were subsequently measured. Compared to the Control group, LA-V4 supplementation did not alter the concentrations of CREA and UA, and the TG level was similarly maintained within a normal baseline (Fig. 2C, *p* > 0.05). This indicates that LA-V4 safely modulates systemic physiological functions without imposing additional metabolic or excretory burdens on the liver and kidneys. Immunoglobulins play a pivotal role in the host defense system and serve as classic indicators for evaluating immune function. Further quantitative analysis revealed that LA-V4 supplementation significantly elevated the overall levels of immunoglobulins in broilers. Specifically, the concentrations of IgM, sIgA, and IgG in the LA group were markedly upregulated compared to those in the Control group (Fig. 2D, *p* < 0.001). Collectively, these findings illustrate that early dietary supplementation with LA-V4 exerts no pathological damage to broilers, while effectively facilitating the synthesis and accumulation of immunoglobulins, thereby enhancing the systemic immune function of the host.

### Effects of dietary LA-V4 supplementation on gut microbiota in broilers

**Fig. 3:**
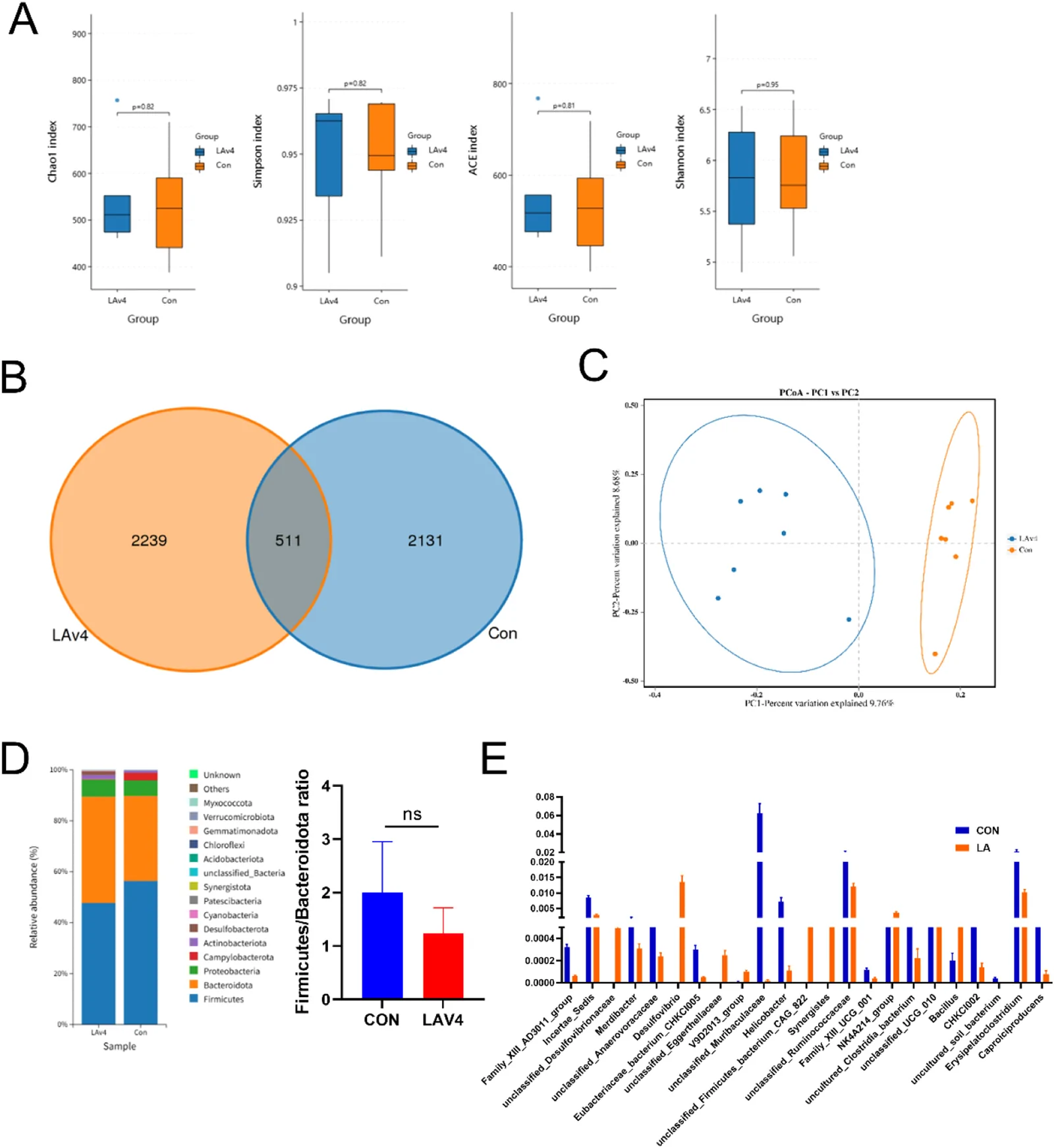
Effects of LA-V4 supplementation in the diet on the colonic microbiota in healthy broilers. **(A)** Alpha diversity indices (Chao1, Simpson, ACE, and Shannon). **(B)** Venn diagram of OTUs. **(C)** Beta diversity evaluated by PCoA. **(D)** Relative abundance at the phylum level and Firmicutes/Bacteroidota ratio. **(E)** Relative abundance of significantly altered bacteria at the genus level based on Metastats analysis.

To assess the modulatory impact of dietary LA-V4 on the gut microecosystem, the microbial composition and diversity of broiler cecal contents were comprehensively profiled via high-throughput 16S rRNA gene sequencing. Alpha diversity analysis showed that LA-V4 supplementation neither reduced the diversity of the microbiota (Simpson index and Shannon index, *p* > 0.05), or alter the community diversity (Choa1 index and ACE index, *p* > 0.05, Fig. 3A). Venn diagram analysis revealed that both groups shared 511 Operational Taxonomic Units (OTUs), while LA-V4 supplementation enriched with 2239 unique OTUs, indicating that LA-V4 intervention promotes the recruitment of specific microbial populations (Fig. 3B). Principal coordinate analysis (PCoA) based on β-diversity revealed that the Control and LA groups formed completely distinct and non-overlapping clusters in spatial distribution, further confirming that LA-V4 supplementation significantly reshaped the overall community structure of the cecal microbiota in healthy broilers (Fig. 3C). Taxonomic composition analysis at the phylum level revealed that Firmicutes and Bacteroidota constituted the dominant microbial communities in the ceca of both groups. Compared to the Control group, the relative abundance of Bacteroidota in the LA group was significantly elevated. Furthermore, LA-V4 supplementation effectively decreased the relative abundance of the potentially pathogenic Campylobacterota phylum in healthy broilers. We subsequently evaluated the Firmicutes-to-Bacteroidetes (F/B) ratio. Although the reduction did not achieve statistical significance, LA-V4 intervention visibly drove a downward shift in this index relative to the unchallenged control group (Fig. 3D). At the genus level, early intervention with LA-V4 induced substantial successions in specific bacterial taxa. Specifically, the abundances of *Bacteroides* and the *Rikenellaceae RC9* gut group were significantly upregulated in the LA group. Conversely, the abundances of the primary foodborne pathogen Campylobacter and the barrier-damaging *[Ruminococcus]_torques_group* were notably suppressed. Further t-test analysis of inter-group species abundance at the genus level using Metastats software confirmed that LA-V4 intervention profoundly reshaped the cecal microbial community architecture (Fig. 3E). This reshaping was primarily characterized by the depletion of potential pathogens and the selective enrichment of specific functional bacteria. Specifically, the significantly downregulated genera in the LA group included *Erysipelatoclostridium*, the potentially pathogenic *Helicobacter* and *Merdibacter*, as well as unclassified Muribaculaceae and Ruminococcaceae. Concurrently, the significantly enriched genera comprised classical probiotic taxa such as Bacillus, the butyrate-producing NK4A214_group, unclassified_Firmicutes_bacterium_CAG_822, and Desulfovibrio. These directional fluctuations of core taxa collectively exert a multidimensional positive impact on the intestinal microecological homeostasis and physiological health of broilers.

### LA-V4 pretreatment enhances resistance to *Salmonella Typhimurium* infection

**Fig. 4:**
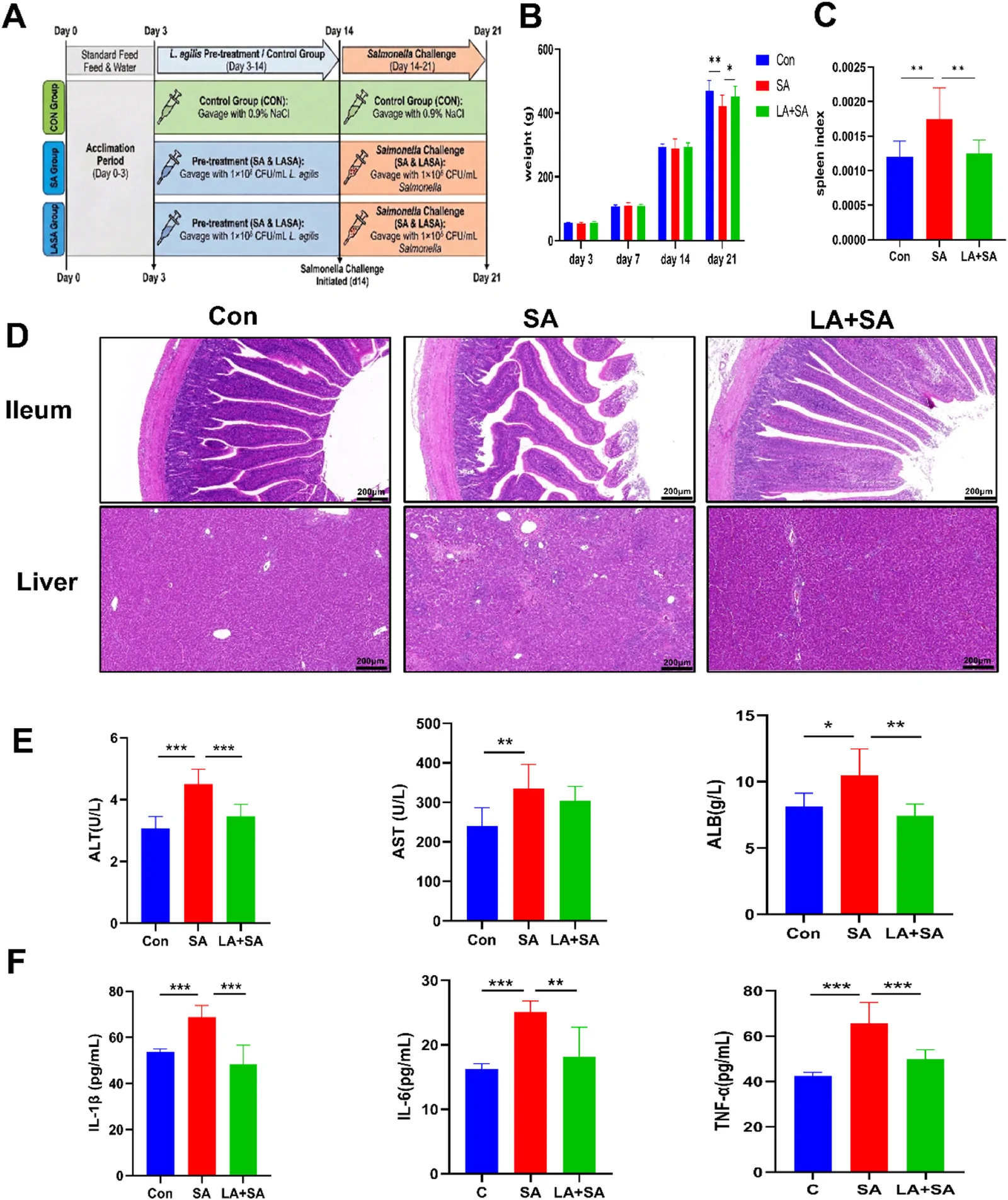
Protective effects of L.A-V4 against *S.* Typhimurium infection in broilers. **(A)** Schematic of the experimental design. **(B)** Body weight. **(C)** Spleen index. **(D)** Representative H&E staining of jejunum and liver tissues. **(E)** Serum levels of liver function markers (ALT, AST, and ALB). **(F)** Serum levels of inflammatory cytokines (IL-1β, IL-6, and TNF-α). Data are presented as mean ± SD. *p < 0.05, **p < 0.01, ***p < 0.001.

*Salmonella* is widely recognized as a leading enteric pathogen causing severe diarrhea in broiler production, which can readily breach the intestinal mucosal barrier and subsequently invade deep host tissues (Demircioglu, et al., 2024). To systematically evaluate the protective effect of preventive supplementation with LA-V4 supplementation on intestinal function, we established a diarrhea model using a *Salmonella* Typhimurium challenge as shown in Fig. 4A. The experiment design included three groups: the Control group, SA group and LASA group. The results demonstrated that prophylactic LA-V4 supplementation alleviated the clinical signs associated with *S*. Typhimurium infection. Compared with the SA group, broilers in the LASA group exhibited significantly higher body weights, although their body weights remained lower than those of the Control group (*p* < 0.05, Fig. 4B). Moreover, the significant increase in the spleen index caused by *Salmonella* infection was effectively prevented by dietary supplementation of LA-V4 (*p* < 0.01, Fig. 4C). Histological evaluation revealed that *Salmonella*-infected group exhibited severe morphological destruction in the jejunum, characterized by shortened, blunted and fused villi, as well as epithelial shedding and damage. However, prophylactic LA-V4 supplementation ameliorated these intestinal pathological lesions. Furthermore, *Salmonella* infection induced distinct hepatic injury, primarily manifested as disorganized hepatic cords and pronounced focal inflammatory cell infiltration. In stark contrast, the liver architecture in the LA-V4 supplemented group was remarkably preserved, with regular hepatocyte arrangement restored and inflammatory infiltration substantially reduced. Subsequently, we evaluated the levels of key hepatic functional indicators in the serum (Fig. 4D). Consistent with the histological observations, *Salmonella* challenge induced an abnormal elevation in the serum concentrations of ALT, AST, and ALB in broilers; conversely, prophylactic supplementation with LA-V4 significantly reversed this pathological progression (Fig. 4D, *p* < 0.01). Next, we measured serum cytokine levels by ELISA and found that *Salmonella* treatment significantly increased the levels of pro-inflammatory cytokines IL-1β, IL-6 and TNF-α (Fig. 4E) in the broiler serum. Furthermore, compared with the SA group, the LASA group showed a prominently reduction in pro-inflammatory of IL-1β and TNF-α (*p* < 0.001), and a significant reduction in IL-6 (*p* < 0.01). Collectively, these findings indicate that prophylactic LA-V4 intervention effectively suppresses the elevated host inflammatory tone provoked by *Salmonella* infection.

### Effects of LA-V4 pretreatment on intestinal barrier function and autophagy in broiler

**Fig. 5:**
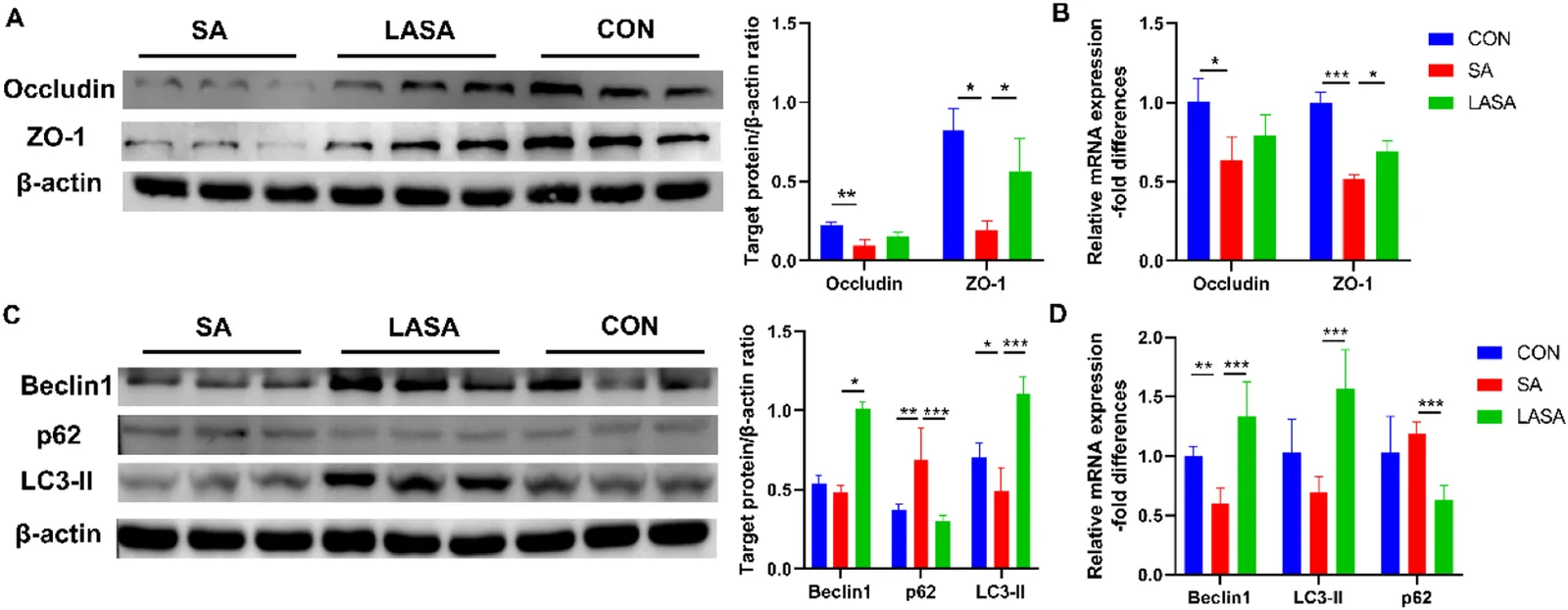
Effects of L.A-V4 on intestinal barrier integrity and hepatic autophagy in *S.* Typhimurium-infected broilers. **(A)** Western blot analysis and protein quantification of intestinal tight junction proteins (Occludin and ZO-1). **(B)** Relative mRNA expression of Occludin and ZO-1 in intestinal tissues. **(C)** Western blot analysis and protein quantification of hepatic autophagy-related proteins (Beclin1, p62, and LC3-II). **(D)** Relative mRNA expression of Beclin1, LC3-II, and p62 in hepatic tissues. Data are presented as mean ± SD. *p < 0.05, **p < 0.01, ***p < 0.001.

To further elucidate the restorative effects of prophylactic LA-V4 supplementation on the intestinal epithelial mechanical barrier, we evaluated the mRNA and protein expression profiles of critical tight junction proteins (ZO-1 and Occludin) using RT-qPCR and Western blotting. The results showed that *Salmonella* infection markedly downregulated the expression levels of ZO-1 and Occludin at both the transcriptional and translational levels. Supplementation with LA-V4 effectively reversed the infection-induced downregulation of ZO-1 and Occludin (Fig. 5A-B). The liver serves as a critical immunological firewall against the systemic dissemination of enteric pathogens. Autophagy acts as a highly conserved innate defense mechanism, playing an indispensable role in the clearance of intracellular bacteria. Following our observation of *Salmonella*-induced hepatic damage, we investigated the underlying autophagic responses. Results revealed that *Salmonella* infection markedly suppressed Beclin1 and LC3-II levels while triggering a substantial accumulation of p62 (Fig. 5C-D), indicative of impaired autophagic flux. Conversely, LA-V4 pretreatment successfully abolished this blockade, restoring normal autophagic flux and thereby fortifying hepatic defense against *Salmonella*.

### Effects of LA-V4 on the cecal microbiota composition in *Salmonella*-infected broilers

**Fig. 6:**
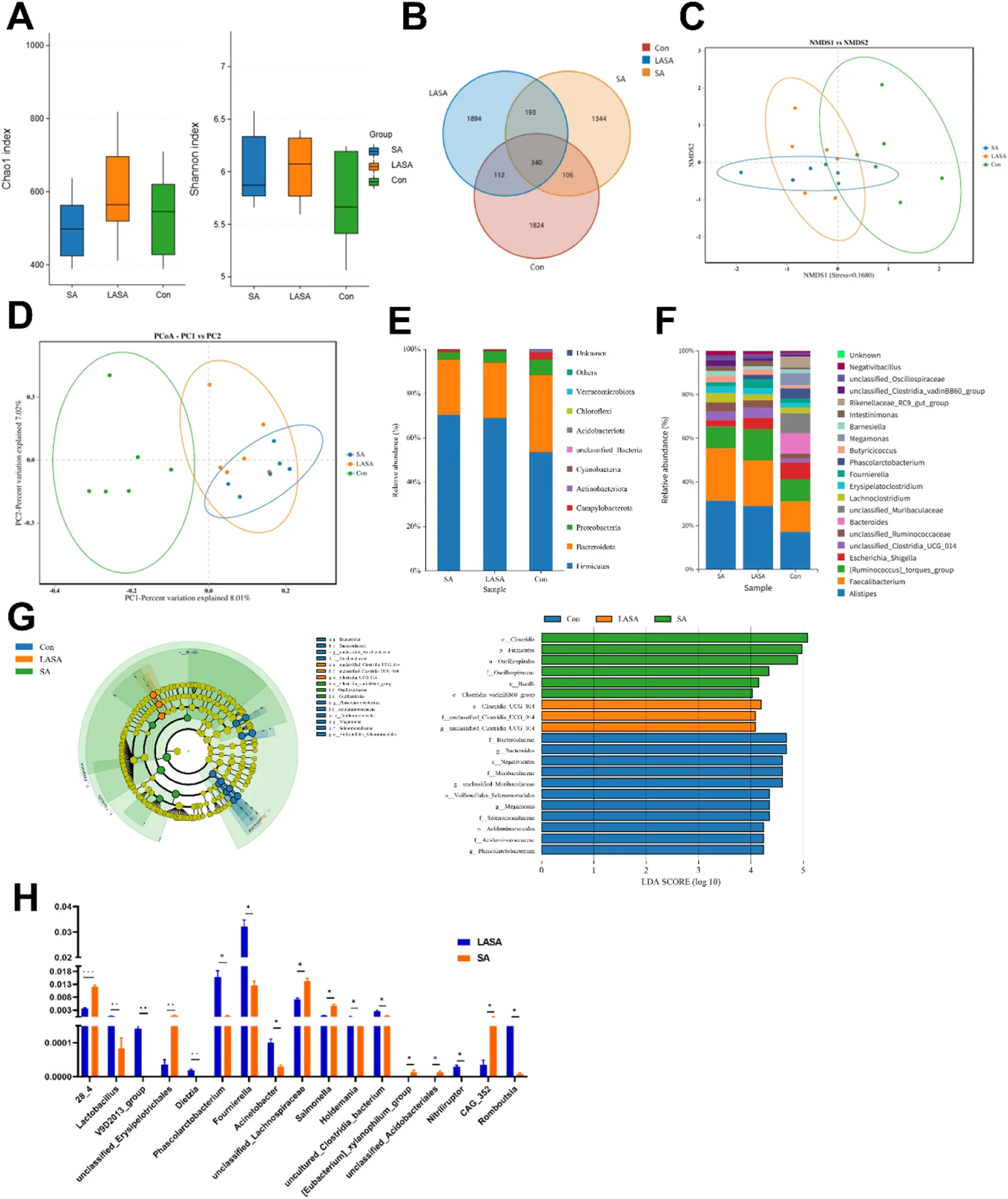
L.A-V4 modulates the cecal microbiota in *S.* Typhimurium-infected broilers. **(A)** Alpha diversity indices (Chao1 and Shannon). **(B)** Venn diagram of OTUs. **(C)** NMDS and **(D)** PCoA plots of beta diversity. **(E)** Relative abundance at the phylum level. **(F)** Relative abundance at the genus level. **(G)** LEfSe analysis identifying specific microbial biomarkers (cladogram and LDA scores). **(H)** Relative abundance of significantly altered bacteria at the genus level based on Metastats analysis between the SA and LASA groups. Data in (H) are presented as mean ± SD. *p < 0.05.

To further characterize the effects of prophylactic LA-V4 supplementation on the cecal microbiota during *S*. Typhimurium infection, we analyzed changes in microbial community composition. Alpha diversity analysis showed that compared with the *Salmonella*-infected group, LA-V4 supplementation prominently increased the Shannon index and Chao1 index (Fig. 6A), indicating that LA-V4 supplementation notably enhanced the diversity and richness of the cecal microbiota. Venn diagram analysis (Fig. 6B) showed that three treatment group shared 340 OTUs, the numbers of unique OTUs identified in the CON, SA and LASA groups were 1824,1344 and 1894, respectively. Both NMDS and PCoA results further unraveled that pronounced structural shifts and clear inter-group differences within the overall cecal microbiome of the broilers (Fig. 6C-D). The Control and LA-V4 groups exhibited distinct and tight clustering, whereas the SA group was relatively dispersed, highlighting profound shifts in the microbial community structure. Taxonomic profiling at the phylum and genus levels further delineated the compositional shifts in the cecal microbiota among the treatment. At the phylum level, Firmicutes and Bacteroidota dominated the microbial community across all groups. Compared to the Control group, *Salmonella* infection induced an apparent expansion of Firmicutes and a concurrent reduction in Bacteroidota (Fig. 6E). More pronounced taxonomic divergences were observed at the genus level (Fig. 6F). The SA group was characterized by an abnormal enrichment of *Alistipes* and *Faecalibacterium*, alongside a severe depletion of *Bacteroides*. Notably, prophylactic LA-V4 supplementation partially restrained the overproliferation of *Alistipes* and distinctly elevated the relative abundance of the *[Ruminococcus]_torques_group* compared to the SA group. These taxonomic findings suggest that LA-V4 mitigates *Salmonella*-induced dysbiosis by modulating the proportions of specific key genera. LEfSe analysis further revealed the differential characteristics of the cecal microbiota among the three broiler groups. The results indicated that *Salmonella* infection induced profound structural alterations in the intestinal microbial community, with specific taxa, including the *Clostridia* vadinBB60 group, *Oscillospiraceae*, and *Alistipes massiliensis*, displaying marked abnormal enrichment in the SA group. Conversely, prophylactic LA-V4 supplementation attenuated the infection-associated enrichment of these taxa, as evidenced by their significantly lower relative abundances in the LASA group than in the SA group. Metastats analysis was subsequently employed to evaluate the specific taxonomic differences at the genus level between the SA and LASA groups. The data demonstrated that the relative abundance of *Salmonella* in the cecum was significantly lower in the LASA group compared to the SA group (*p* < 0.05). These findings indicate that LA-V4 pretreatment was associated with a lower relative abundance of *Salmonella*-assigned sequences in the cecal microbiota. Concurrently, the relative abundances of potential opportunistic pathogens, such as *Fournierella* and unclassified *Lachnospiraceae*, were significantly elevated in the SA group, whereas this trend was substantially suppressed in the LASA group. Collectively, these findings indicate that prophylactic LA-V4 supplementation strongly preserves intestinal microbial homeostasis during *Salmonella* infection by antagonizing pathogen colonization and rectifying the dysbiosis of specific commensal taxa.

## Discussion

*Salmonella* Typhimurium, as one of the most common enteric pathogens in poultry farming, has a significant negative impact on broiler production. In addition, its zoonotic spread via the food chain by contaminated poultry-contained foods poses a significant risk to human health, and hence it is a key causative agent of human salmonellosis. (Perry and Yousef, 2012; Shaji, et al., 2023). Extensive antibiotic application has incited growing public apprehension over broiler carcass quality and product safety, driving the urgent need for viable antibiotic alternatives (Wang, et al., 2025b). Recently, the isolation of probiotics from naturally resilient animal hosts has emerged as a promising strategy for developing dietary interventions against enteric pathogenic infections. Based on our previous isolation of *Ligilactobacillus agilis* LA-V4 from the feces of apparently healthy vultures (Li, et al., 2026), the present study demonstrated that early prophylactic supplementation with LA-V4 significantly alleviated *S*. Typhimurium-induced intestinal injury in broilers. The protective effects of LA-V4 may be associated with the modulation of hepatic autophagy-related processes, remodeling of the gut microbiota, and reinforcement of the intestinal mucosal barrier.

Before any novel probiotic candidate can be considered for practical application, evaluating its *in vivo* safety is an indispensable prerequisite (Binda, et al., 2020). Therefore, the present study initially assessed the effects of early LA-V4 dietary intervention in healthy broilers. The results demonstrated that LA-V4 supplementation induced no adverse clinical signs or histopathological lesions, while effectively maintaining normal growth performance. Interestingly, LA-V4 reduced the liver index significantly as compared to the control group. Follow-up histological and serum biochemical tests indicated that alanine aminotransferase (ALT) levels were moderately reduced, and the concentration of aspartate aminotransferase (AST) and albumin (ALB) were normal. The literature available suggests that subclinical hepatic metabolic stress is common in modern commercial broilers because of the high growth rates. (Estévez, 2015). Taken together, these findings suggest that dietary LA-V4 supplementation may contribute to maintaining hepatic metabolic homeostasis in rapidly growing broilers. This finding is highly consistent with previous studies reporting similar hepatoprotective effects following probiotic interventions (Wang, et al., 2025a). In addition, the concentration of major immunoglobulins (IgM, IgG and sIgA) was dramatically increased by LA-V4, which supports the ability of the probiotic to enhance the immune systems of the host, both systemically and at mucosal sites, which was previously reported in regards to probiotic-based immunomodulation (Zhao, et al., 2024). Concerning intestinal health, histopathological analyses revealed that LA-V4 supplementation did not induce any adverse alterations in intestinal morphology. Quantitative morphometric analysis further showed that LA-V4 supplementation significantly reduced jejunal crypt depth and increased the villus height-to-crypt depth ratio, whereas villus length remained unchanged. Although reduced crypt depth alone cannot be interpreted as an unequivocally beneficial alteration, the increased villus height-to-crypt depth ratio and the absence of histopathological abnormalities suggest that LA-V4 supplementation did not impair overall intestinal architecture. Additional examination of the intestinal tight junction proteins showed that the expression of ZO-1 and Occludin in the jejunum increased but not significantly. Microbiome analysis revealed that, although LA-V4 did not have a significant effect on overall alpha diversity, it was able to change the structure of the cecal microbial community. In particular, it enriched significantly core short-chain fatty acid (SCFA)-producing taxa, such as *Bacillus*, v9d2013_group, and unclassified_Firmicutes_bacterium_CAG_822 (Fusco, et al., 2023). Given that SCFAs are pivotal for maintaining gut microbiota homeostasis (Hays, et al., 2024), the following initial results confirm that LA-V4 is extremely biosafe and plays an active role in the preservation of homeostasis in the intestines, the reduction of the metabolic load, and immune response regulation. Substantial evidence has shown that *S*. Typhimurium infection has a devastating impact on the growth performance of broilers (Choi, et al., 2022; Wang, et al., 2023). When the pathogen breaks through the intestinal barrier, the pathogen is rapidly translocated throughout the whole body and invades the liver and the spleen causing a significant amount of parenchymal damage that eventually occurs as hepatosplenomegaly (Perez-Toledo, et al., 2025; Wang, et al., 2024). As these findings suggest, our experiment showed that *S*. Typhimurium challenge caused significant growth retardation, increased spleen indices, and apparent hepatic damage in broilers. Interestingly, these pathological changes were reversed by prophylactic intervention with LA-V4. The anti-inflammatory properties of *Lactobacillus* species have been extensively reported in recent years. As an example, in a murine model of Escherichia coli-induced diarrhea, Feng et al. demonstrated that *Lactobacillus agilis* SNF7 had excellent anti-inflammatory effects (Feng, et al., 2024), as Hou et al. showed excessive inflammatory reactions in obesity models were suppressed by *Lactobacillus* supplementation. (Hou, et al., 2025). In our current study, serum cytokine profiling revealed that prophylactic LA-V4 supplementation significantly abrogated the systemic inflammatory response triggered by the infection. Synthesizing these findings, we postulate that specific *Lactobacillus* strains may employ conserved immunomodulatory mechanisms to efficiently blockade infection-induced inflammatory cascades.

The intestinal barrier constitutes the first line of defense against foodborne bacterial infections. In the present study, *S*. Typhimurium infection caused damage to the intestinal epithelial villi and mucosa of the host, resulting in the disruption of jejunal structural integrity. Remarkably, prophylactic supplementation with LA-V4 significantly ameliorated these architectural lesions. This observation aligns closely with the findings of Li et al. (Li, et al., 2023). The stability of the intestinal physical barrier relies heavily on tight junction (TJ) proteins. Previous studies have indicated that the downregulated expressions of these proteins in enteric diseases typically signifies impaired barrier function and elevated intestinal permeability, thereby creating a permissive environment for the invasion of external noxious stimuli (Chelakkot, et al., 2018; Kuo, et al., 2022). Previous studies have well documented that *S.* Typhimurium severely compromises the structural and functional integrity of intestinal tight junction proteins (Boyle, et al., 2006). Conversely, dietary supplementation with *Lactobacillus* can significantly reverse this damage, thereby restoring the overall integrity of the intestinal barrier (Lépine, et al., 2018; Wang, et al., 2018). In the present study, *S.* Typhimurium infection damaged the intestinal epithelial villi and mucosa, disrupted jejunal structural integrity, and markedly suppressed the expression of the critical tight junction proteins ZO-1 and Occludin in broiler jejunal tissue, whereas LA-V4 supplementation significantly reversed these alterations. Although preserving intestinal barrier integrity is the first line of defense, during severe infection, *S.* Typhimurium can breach the damaged mucosa and undergo systemic translocation to the liver via the portal circulation (Shao, et al., 2013; Yin, et al., 2024). As the liver serves as the central defense organ for intercepting gut-derived pathogens and preventing their systemic dissemination, its intrinsic anti-infective mechanisms are of paramount importance. Autophagy, a highly conserved intracellular degradation and defense process, contributes to the indispensable recognition and elimination of invading intracellular bacteria (Luis, et al., 2022). Previous studies have demonstrated that intracellular *S.* Typhimurium actively suppresses host autophagy to facilitate its survival and subsequent dissemination, at immune evasion strategy likely dependent on expression of virulence effectors such as *Spv* B and *Spv* C (Wu, et al., 2020; Zhou, et al., 2021). Restoring the impaired autophagic flux is considered a highly effective therapeutic avenue to alleviate *S.* Typhimurium infection (Liu, et al., 2018; Tan, et al., 2020). In the present study, *S.* Typhimurium infection significantly decreased the hepatic levels of LC3-II and Beclin1 while increasing p62 accumulation. The coordinated alterations in these autophagy-related proteins provide supportive evidence that *S.* Typhimurium infection disrupted hepatic autophagy-related processes and may have impaired autophagic degradation. Notably, prophylactic supplementation with LA-V4 markedly ameliorated these infection-associated alterations. These findings suggest that modulation of hepatic autophagy-related processes may contribute, at least in part, to the hepatoprotective effects of LA-V4. However, because LC3-II, Beclin1, and p62 were evaluated under steady-state conditions, these measurements do not fully capture the dynamic nature of autophagic flux. Therefore, the present findings do not conclusively establish either the blockade or restoration of autophagic flux, and further dynamic flux assays are required to clarify this process.

Further profiling of the microbial community revealed that *S.* Typhimurium infection severely altered the host's gut microbiota architecture, consistent with previous reports (Yi, et al., 2023). Although no significant changes in α-diversity were observed, the distinct separation in β-diversity indicated an overall ecological shift induced by the challenge. At the phylum level, SA group exhibited a higher mean relative abundance of Firmicutes and lower mean relative abundances of Bacteroidota, Proteobacteria, and Campylobacterota than the control group. LA-V4 supplementation induced relatively modest phylum-level changes, characterized by a slight decrease in Firmicutes and an increase in Proteobacteria toward the control profile, whereas Bacteroidota remained broadly comparable to that in the infection group and Campylobacterota did not show an evident recovery. Hu et al. (2022) similarly reported that *S*. Enteritidis infection decreased the overall abundance of Proteobacteria but increased *Salmonella* in chickens (Hu, et al., 2022), whereas Thiam et al. (2022) observed a higher abundance of Proteobacteria in chickens with greater resistance to infection (Thiam, et al., 2022). A previous study from our laboratory using the same *S*. Typhimurium strain also observed a lower mean abundance of Proteobacteria in infected broilers than in uninfected controls (Wang, et al., 2026). In the present study, LA-V4 shifted the relative abundance of Proteobacteria toward the control profile while reducing *Salmonella*-assigned sequences, suggesting a taxon-specific restructuring within Proteobacteria rather than a general expansion of potentially pathogenic members. The genus-level Metastats results further supported this interpretation.

Previous studies have demonstrated that *S*. Typhimurium infection significantly reshapes the avian gut microecology, and infection-associated inflammatory conditions may favor the expansion of specific Firmicutes members, such as Lachnospiraceae and Erysipelotrichales (Khan and Chousalkar, 2020; Zheng, et al., 2024). Genus-level Metastats and LEfSe analyses were consistent with this pattern, showing increased relative abundances of Firmicutes-related taxa, such as unclassified_Lachnospiraceae and unclassified_Erysipelotrichales, in the cecum. Conversely, prophylactic LA-V4 administration partially reversed the increased relative abundances of these taxa. Moreover, LA-V4 pretreatment was associated with a significantly lower relative abundance of *Salmonella-*assigned sequences in the cecal microbiota. LA-V4 also increased the relative abundance of Lactobacillus and several putative short-chain fatty acid (SCFA)-producing or SCFA-associated taxa, including v9d2013_group, Phascolarctobacterium, and Fournierella. Phascolarctobacterium has been reported to produce acetate and propionate (Wu, et al., 2017). Research indicates that gut-derived propionate can directly suppress *Salmonella* virulence by targeting the LuxS/AI-2 quorum-sensing system(Zhang, et al., 2026). Furthermore, SCFAs can modulate the expression of pathogen invasion-related genes while serving as essential energy substrates to facilitate intestinal mucosal repair(Durant, et al., 2000; Hays, et al., 2024). More importantly, SCFAs may also participate in gut–liver communication. Multiple *in vivo* and *in vitro* studies have demonstrated that SCFAs can modulate hepatic function and attenuate liver injury(Lu, et al., 2025; Miao, et al., 2025). As demonstrated by Iannucci et al. (2016), chronic depletion of the murine gut microbiota with antibiotics reduced basal hepatic autophagy, whereas propionate and butyrate directly regulated autophagic flux in hepatic cells through a mechanism involving PPARγ-dependent UCP2 expression, reduced intracellular ATP levels, and AMPK activation.(Iannucci, et al., 2016). In the present study, LA-V4 supplementation concurrently enriched several putative SCFA-producing taxa in the cecal microbiota and ameliorated the infection-associated alterations in hepatic LC3-II, Beclin1, and p62 levels. The parallel changes observed in the intestinal microbiota and hepatic autophagy-related proteins raise the possibility that LA-V4-induced microbial remodeling may influence hepatic autophagy-related processes through microbiota-derived metabolite signaling along the gut–liver axis. In particular, the enrichment of putative SCFA-producing taxa may alter intestinal metabolic output, which could subsequently contribute to the regulation of hepatic autophagy-related activity. Nevertheless, because SCFA concentrations and dynamic autophagic flux were not directly measured, this proposed relationship remains a literature-supported mechanistic hypothesis.

Taken together, we postulate that the protective effects of LA-V4 against *S*. Typhimurium infection may be partially associated with its modulation of the impaired gut microbiota. By promoting the enrichment of putative SCFA-producing taxa, LA-V4 may contribute to the restriction of local pathogen proliferation and the repair of the intestinal barrier. Based on previous evidence regarding SCFA-mediated gut–liver communication, we further hypothesize that microbiota-derived SCFAs may participate in the regulation of hepatic autophagy-related processes and thereby contribute to the alleviation of infection-induced liver injury. However, SCFA concentrations in the intestinal contents, portal blood, and peripheral circulation were not directly quantified in the present study. Therefore, the proposed microbiota–SCFA–liver autophagy pathway should be regarded as a literature-supported hypothesis rather than a causal mechanism demonstrated by our data. Future studies should incorporate targeted SCFA profiling, dynamic autophagic flux assessment, and functional intervention experiments to validate this proposed pathway.

An additional limitation of the present study is that the baseline *Salmonella* status of the birds was not directly verified by bacteriological culture or PCR before the experimental challenge. Although no *Salmonella*-assigned sequences were detected in the unchallenged control birds from the safety experiment by 16S rRNA sequencing, this finding cannot substitute for targeted pre-challenge screening. Furthermore, although the single-dose *S*. Typhimurium challenge model successfully induced typical infection-associated alterations and enabled evaluation of the protective effects of LA-V4, it may not fully represent the dynamic process of bacterial persistence and long-term host–pathogen interactions. Future studies should incorporate targeted microbiological detection and longitudinal monitoring of bacterial burden to further clarify the protective mechanisms of LA-V4.

## Conclusions

In summary, this study demonstrates that prophylactic supplementation with *Ligilactobacillus agilis* LA-V4 alleviated *S*. Typhimurium-associated intestinal and hepatic injury in broilers. LA-V4 improved intestinal barrier integrity by increasing the expression of tight junction proteins and modulated the cecal microbial community, as reflected by a lower relative abundance of *Salmonella*-assigned sequences and increased relative abundances of several putative SCFA-producing taxa. In the liver, LA-V4 reversed the infection-associated alterations in LC3-II, Beclin1, and p62, providing supportive evidence for the modulation of hepatic autophagy-related processes. Collectively, these findings support the probiotic potential of LA-V4 in maintaining intestinal barrier function, modulating the gut microbiota, and alleviating hepatic injury during *S*. Typhimurium infection. However, the contribution of SCFAs and the dynamic changes in autophagic flux require further validation through targeted metabolomic and functional analyses.

## Author contribution

Chuxian Quan conducted the experimental work, analyzed and interpreted the data, and drafted the manuscript. Siyuan Li participated in the experimental work, analyzed and interpreted the data, and contributed to drafting the manuscript. Ziyang Wang conducted experimental investigations and contributed to data analysis and interpretation. Rui Zhao contributed to the analysis and interpretation of the experimental findings. Muhammad Farhan Rahim contributed to the development of the experimental procedures and the analysis and interpretation of the data. Mengdi Zhang participated in the experimental investigation and contributed to data analysis and interpretation. Chao Jin contributed to the analysis and interpretation of the experimental findings. Haofang Yuan contributed to data organization, analysis, and interpretation. Yan Li contributed to the study design and the analysis and biological interpretation of the experimental findings. Shan Wu participated in the experimental investigation and contributed to the analysis and interpretation of the study findings. Saisai Gong contributed to the analysis and interpretation of the experimental findings. Quan Mo contributed to the study design and the analysis and biological interpretation of the experimental findings. Jiakui Li conceived the study and provided intellectual guidance for the development and interpretation of the research.

## Declaration of competing interest

The authors declare no conflicts of interest.

## Disclosures

The final version of the manuscript is read and approved by all authors who are going to submit it to Poultry Science. The work is original and has never been published in its entirety or part thereof or is under consideration in another source.

## Data Availability

Data will be available on request.
